# Test@work: evaluation of workplace HIV testing for construction workers using the RE-AIM framework

**DOI:** 10.1186/s12889-021-11739-z

**Published:** 2021-09-24

**Authors:** Wendy Jones, Sarah Somerset, Catrin Evans, Katharine Whittingham, Matthew Middleton, Holly Blake

**Affiliations:** 1 School of Health Sciences, University of Nottingham, Nottingham, UK; 2 School of Medicine, University of Nottingham, Nottingham, UK; 3 NIHR Nottingham Biomedical Research Centre, Nottingham, UK

**Keywords:** HIV testing, Workplace, Health promotion, Construction, Health checks, Occupational health, RE-AIM

## Abstract

**Background:**

Community testing for HIV can reach previously untested populations but is rarely offered in workplaces. Targeting the construction sector could reach workers from high risk populations.

**Methods:**

The RE-AIM framework was used to evaluate Test@Work, a workplace HIV testing intervention for construction workers implemented at 21 events (10 companies) in the UK.

Test@Work had three components: 1) an online health toolkit to inform managers about health screening and HIV testing; 2) general health checks; and 3) opt-in HIV consultation and testing. Quantitative data were collected using registration and exit questionnaires with workers (*n* = 426) and pre/post-event questionnaires with managers (*n* = 15), with qualitative analysis of free text responses.

**Results:**

*Reach* 426 individuals had health checks. Participants were broadly representative of the UK construction workforce, but with a higher proportion of permanent workers. Most workers reported being in good health but also believed their work had an adverse impact on their health. *Effectiveness*: 97% of health check participants opted to have a consultation about sexual health (*n* = 413) and 82% had an HIV test (*n* = 348), of whom 78% had not previously been tested. All HIV tests were non-reactive. HIV testing at work was considered acceptable by most participants. Participants reported learning new things about their health (74%), said they would make changes as a result (70%) and felt confident of success (median score 8/10). *Adoption:* Recruitment of companies was challenging and time consuming. Seven of the participating companies were very large, employing over 1000 workers, which is atypical of construction generally. *Implementation:* All events were completed as planned and were considered successful by all parties. *Maintenance:* All managers would arrange further events if they were offered them. Six managers incorporated sexual health awareness into their health programmes, but this was not possible for many as health agendas were set centrally by their organisations.

**Conclusions:**

Opt-in HIV testing, when embedded within a general health check, has high uptake and acceptability in the UK construction sector, and reaches individuals at risk for HIV who may not otherwise attend for testing. Cost-effectiveness of this approach is yet to be determined.

**Trial registration:**

ClinicalTrials.gov Identifier: NCT04292002.

**Supplementary Information:**

The online version contains supplementary material available at 10.1186/s12889-021-11739-z.

## Background

The last twenty years have seen substantial developments in the treatment and prevention of HIV. Worldwide, 26 million people are receiving lifelong ART (antiretroviral therapy) and the rate of new infections is falling in many countries. Within the United Kingdom (UK), 97% of those who have been diagnosed with the illness are receiving treatment. Viral suppression has been achieved in 97% of those being treated so that they are no longer considered infectious and their life expectancy and quality of life are close to normal [1]. There have also been substantial improvements in diagnosis rates, particularly for men who have sex with men (MSM). However, an estimated 19% of those living with the disease worldwide do not know their HIV status. Within the UK, there remain an estimated 7500 undiagnosed cases and 43% of cases are diagnosed late, resulting in higher mortality and an ongoing risk of transmission to others. Late diagnosis is more common amongst men who are heterosexual and older [2, 3]. Additionally, both HIV prevalence and late diagnosis in the UK are higher amongst those with black ethnicity and those born in countries with a high prevalence [4]. More than 50% of infections are acquired post-migration for these populations [5, 6] highlighting the need for ongoing education and effective testing programmes.

Increasing testing uptake, particularly for those at greatest risk, has been identified as the single most important action to improve the management of HIV in the UK [7]. Advances in recent years to support this have included self-sampling and self-testing kits, shown to have high sensitivity and specificity, high effectiveness (particularly amongst black and Asian men in the UK) and good acceptability [3, 7, 8]. There have also been developments in the variety of settings which are used for testing. For example, primary health care settings such as general practices (GP) in high prevalence areas are encouraged to test routinely; sexual health clinics are advised to test those who attend for other services, as often as every three months for those at greatest risk [1]. Community-based testing has been introduced in many countries, often targeting high risk populations e.g., through stalls at Gay Pride events, bars and saunas, also in outreach clinics away from main health care centres [9–13]. These events are typically more expensive and report lower rates of positive results than more traditional testing routes but have successfully identified cases which may have been missed through normal channels.

Community testing has also been conducted in workplace settings, but these interventions have mostly been in sub-Saharan Africa [11]. Programmes to test, educate and address HIV-related stigma are reported, for example in South Africa [14], Uganda [15] Zambia [16] and Zimbabwe [17]. These studies have focused particularly in sectors with male dominated populations and a high proportion of migrant workers, such as construction and security. Such programmes are challenging; considerable efforts are needed to address difficulties such as the stigma associated with a positive diagnosis, fear of compromised confidentiality and low perceived HIV risk [18, 19]. Yet, workplace interventions have shown to be an important route for testing in populations with high HIV prevalence [17, 20], risky lifestyle behaviours and low access to health care [19, 21, 22]. Worksite HIV testing programmes have demonstrated the particular importance of accessibility and convenience for HIV testing with these hard-to-reach populations [17].

The potential for workplace HIV testing programmes outside of Africa has largely been overlooked. Systematic reviews by Thornton et al. [12] and Croxford et al. [13] investigated HIV testing in non-healthcare settings in ‘resource-rich’ countries and the European Union (EU)/European Economic Area (EEA) respectively, but did not identify any workplace testing. However, recent studies have identified that HIV testing in UK workplaces, when combined with general health checks, has high uptake and good acceptability [23–25].

Some of the risk factors associated with high prevalence of HIV in African workplaces [21, 26] are paralleled in the UK construction sector. This presents opportunities to detect undiagnosed cases amongst people who might not access testing through other routes. For example, in the UK (like elsewhere) construction work employs many workers from migrant populations [27], as well as transient or travelling workers, living too far from their homes to be able to commute on a daily basis [28–30]. Living away from home increases the likelihood of risky sexual behaviours [21, 22, 31]. Other factors associated with HIV transmission are drug use and high alcohol intake [21], both common in the construction workforce in many countries, including the UK [30, 32–36].

Problematic alcohol and drug use in construction are part of a wider picture of increased health risk in this population. Smoking [37, 38], obesity [38–40], and poor mental health [41–43] are prevalent in the sector. For example, during the construction of the Olympic Park for London 2012, 41% of the workforce were found to be overweight, 28% were obese and 30% had high blood pressure [44]. These health risks, influenced by socioeconomic factors [40, 45–47], add to harm from specific workplace hazards such as noise, vibration, silica dust and hazardous chemicals [48]. As a result, construction workers are at increased risk of early disability or death compared with other populations, with high prevalence of cardiovascular disease, respiratory ill-health and musculoskeletal conditions [49–55]. Beyond individual health impacts, there are financial consequences for workers, since pension changes require them to continue in employment until aged 67 years and beyond [56, 57]. There are substantial costs for construction employers [58] and the premature loss of workers contributes to the growing skill shortages in the UK construction sector [59]. Supportive interventions to help address these factors are therefore important for individual workers and the wider construction community.

The inclusion of wider health checks when offering workplace HIV testing has been recommended as a strategy to increase uptake and reduce the risk of HIV-related stigma [15, 60]. Workplace health checks in construction, therefore, offer on opportunity to address both the potential for undiagnosed HIV and the wider health challenges for this population. Recent studies by Blake et al. [23, 24] suggest that this is a promising approach in the UK, with high workforce acceptability, but no interventions of this nature have targeted the construction workforce specifically.

### RE-AIM

RE-AIM is a framework for planning and evaluating health promotion interventions [61, 62], to ensure they will work in ‘real world’ scenarios. It evaluates not just the effectiveness of an intervention (which can be disproportionately high in a targeted, carefully chosen population) but also the representativeness of the study population and setting. This increases confidence that an intervention will still be effective when disseminated outside the original setting.

The RE-AIM framework [62] consists of five dimensions. *Reach* is a measure of participation, also an assessment of participant characteristics, and whether they are representative of the targeted population. *Effectiveness* assesses the impact of the intervention on individual outcomes, considering both positive and negative consequences. It also considers whether effectiveness varies between sub-groups. *Adoption* examines whether organisations which participate have similar characteristics to those which do not, and what the barriers might be to adoption. *Implementation* can be described in terms of how consistently the programme is delivered; it should also examine how much it costs. Finally, *Maintenance* assesses the long-term effect of a programme after it has been completed.

RE-AIM has evolved over time, with additional criteria identified or added by different authors [63, 64]. Mixed methods are increasingly advocated [63, 65] in place of the purely quantitative approach described in the original model [61]. It has also been acknowledged that pragmatic and less prescriptive approaches to using the model for planning and evaluation can increase its value and uptake in many public health settings, and that priority should be given to those aspects of the tool which are most appropriate for the research question, setting and stakeholders [62].

### Project aims

We aimed to evaluate the delivery of Test@Work, a workplace HIV testing programme in the UK. In Test@Work, HIV testing was incorporated within a wider health check to increase its acceptability and usefulness to participating companies and workers. The research was conducted in the construction sector as its workforce are at high risk of poor health in addition to being at potentially increased risk of HIV. The aims of this study were to use RE-AIM to assess the suitability of the UK construction sector as a location for workplace HIV testing and wider health testing, and to identify learning points for community HIV testing initiatives in the future.

## Methods

### Research design

Test@Work, a free workplace health check programme, was offered to construction companies. The acceptability and perceived usefulness of the programme were assessed using quantitative and qualitative data from questionnaires completed by participating workers and representatives of the construction companies. Test@Work was prospectively registered (ClinicalTrials.gov Identifier: NCT04292002).

### Setting and participants

Participating companies were construction or construction-related organisations, which had one or more sites operating within a designated geographical area in the Midlands, UK. Potential participant companies were identified from public records and contact made by email with a follow-up phone call. For companies which agreed to take part, pre-arranged health check events were scheduled to take place at their premises/worksite. Further details of the recruitment process are discussed in ‘Adoption’ (below).

Within these companies, all workers present on the day(s) of testing were entitled to participate. A worker could be a direct employee, agency worker or a self-employed worker. They might work for the company which had arranged the event; or any of their subcontractors on site (e.g. specific trades or disciplines). Workers were invited to participate by a range of methods including email or personal invitation from their manager or supervisor; poster; or personal invitation by the research team on the day of the event. Workers were generally permitted to attend during their working time, they were not required to use break or lunch times for health checks, although some preferred to. Wherever possible, appointments were booked in advance, but there was also scope for individuals to turn up for an ad-hoc health check at most events.

### Test@work: workplace health check programme

The programme had three components.

#### Online health toolkit

An online health toolkit, “*Test@Work: Creating Healthy workplaces: a toolkit for employers*” was developed. The development and evaluation of this toolkit has been described elsewhere [66]. The toolkit was circulated to all participating companies to increase managers’ understanding of the purpose and value of HIV testing and other health checks. The toolkit was self-directed and took around 60 min to complete.

#### General health checks

All participants were offered a range of general health checks: height, weight, BMI (body mass index), waist/hip measurements, blood pressure, Patient Health Questionnaire-2 item (PHQ-2, mental health check) [67]. Participants could choose which tests they wished to have. Health checks and tailored feedback were based on the *Making Every Contact Count* approach to delivering brief health promotion intervention and used tools from Health Education England [68]. Health check results were noted on a record sheet which each participant took away with them. Where appropriate (e.g. where test results fell outside recommended ranges), they were advised to take this to their GP or pharmacist and seek further advice. They were also given verbal guidance and a written information pack. No health data were kept by the research team. Further detail about the health checks and delivery process are described elsewhere [69].

Health checks were conducted by a team of volunteer healthcare trainees from professional backgrounds including nursing, physiotherapy, medicine and health psychology. Volunteers were trained to perform the tests and supported throughout as part of a programme to enhance interprofessional learning in healthcare education [69].

#### HIV testing and consultation

All participants were offered a consultation, followed by an optional HIV test. If concerns were raised or risks identified in relation to other sexual health topics such as chlamydia, gonorrhoea etc. these were addressed briefly, with onward referral to a sexual health clinic where appropriate. Testing was provided by independent HIV specialists from one of two outreach or charitable organisations that had substantial experience of providing HIV testing in the community and had clinical governance arrangements in place with NHS England Clinical Commissioning Groups (CCGs). Test kits were 4th generation *Alere Determine™ HIV-1/2.* These provide a test result within twenty minutes enabling participants to be given their test results before they left the health check event. Testing was conducted in accordance with the WHO “5 Cs”: Consent, Confidentiality, Counselling, Correct results and Connections [70].

1. Consent: Attending an HIV consultation was an optional element of the health check (attendance at the health check was also voluntary). All individuals who had a consultation were then given further detailed information before they gave informed consent if they wished to proceed to testing.
2. Confidentiality: Testing was conducted by the HIV specialist(s) in a room or location separate from other health checks. Results were given to individuals face to face after test results had developed. No information about individuals or their test results was shared with the health check team or the employer by the HIV specialist. The only information provided to the research team was whether each individual (identified by participant number) had undertaken a test and/or a health consultation; and the total number of reactive/non-reactive test results for the whole series of events.
3. Counselling: All participants were given information about what was being offered and why; the risk factors associated with HIV (sexual practices and number of partners, sex work, drug use, blood transfusion) and participants’ own personal level of risk; and information about the test offered and how results would be given and followed up.
4. Correct results: The test strips used have high clinical sensitivity and specificity and include built-in controls to confirm accurate testing. Test strips were checked to make sure they were in-date. Results were given to each individual before they left the event, with name and date of birth being checked to match results and individual.
5. Connections: a procedure was in place to respond to any reactive tests. UK guidance is for this to follow locally determined pathways. For both partner organisations a reactive test would be followed up with an urgent blood test at a specialist centre, with laboratory analysis. Confirmed positive tests would lead to referral to a treatment location, which could be close to the worker’s home or workplace, according to individual preference. All treatment for HIV is provided free of charge in the UK regardless of an individual’s personal circumstances e.g. immigration or employment status [1]. Access to a helpline for interim support was also available.

### Research data collection

All participating workers were asked to complete two anonymous, brief questionnaires (Additional file 1). Before the event, they were asked for demographic information and to confirm whether they had previously had an HIV test. After the event they were asked about their experience of the event e.g., whether they had learned anything, whether they intended making changes to their health as a result. Most questions were closed, with multiple choice answers, and one question was open (‘is there anything else you would like to tell us?’).

The main contact for each company was asked to complete two questionnaires (Additional file 2). This population are referred to as ‘managers’: they were a mix of site managers, health and safety (H&S) managers and professionals with responsibility for wellbeing. The first questionnaire included details about the company such as the number of workers and the demographics of the working population. The second, completed after the event, asked a series of open questions about their experiences of the event.

Records kept by delivery partners (healthcare volunteers and HIV specialists) indicated which tests were completed for each anonymised individual (Additional file 3). An interview was conducted with the lead researcher who had organised the events, to gather information on the process of recruiting companies to participate and the organisation and implementation of the events.

Informed consent to participate in the research was obtained from all participants. All methods were carried out in accordance with the relevant guidelines.

### Data analysis

Quantitative data were analysed using IBM SPSS statistics V27 (IBM Corp., Armonk, N.Y., USA). All data were categorical or ordinal (age was condensed into four categories). Comparison between groups or between participant data and comparator populations were made using chi-square tests for categorical data (or Fisher’s exact test where indicated) or Mann-Whitney *U* test for continuous/ordinal data e.g. a response on a scale of 1–10 [71].

Qualitative data from free text responses to open questions in the post-event worker and manager questionnaires were uploaded to NVivo 12 Pro, organised by the questions which were being answered. They were coded using an inductive process initially, with codes being created to reflect the thoughts being expressed in the data [72, 73]. Codes were then mapped against the five dimensions of the RE-AIM framework. Comments were coded to more than one node or theme where appropriate [74].

Qualitative and quantitative findings have been combined in this report, using the qualitative outputs to support and add richness to the numerical data set.

### RE-AIM dimensions in this research

Table 1 shows details of indicators and data sources for the RE-AIM dimensions. To examine *Reach* in this paper, the study population is described in terms of their demographic characteristics. They are compared to the target population, i.e. the UK construction workforce. The study population is also described in terms of their health at work, as this reflects the value of targeting this population, and whether they might benefit from health checks and guidance. The primary outcome for *Effectiveness* is the uptake of HIV testing by participants attending general workplace health checks and whether this varies between subgroups. Additional outcomes relate to the uptake of health checks generally, and the perceived impact of these. Indicators of the acceptability and perceived usefulness of HIV testing are also included. To explore *Adoption*, summary data from the participating companies are presented and compared to sector data. Additional information is included about those companies which were approached but did not participate; perceived reasons for non-participation; and the experience of recruiting companies to the study. The focus of *Implementation* is how successful the events were, based on feedback from the managers and workers who were involved in events. Cost information is also presented. *Maintenance* is operationalised as the sustainability of the programme: have organisations made long term changes as a result of this event, and how likely is it that they would participate in future events?

**Table 1:** Operationalisation of RE-AIM Dimensions and indicators used

Indicator	Data source
Reach	
*Who participated, were they representative of the target population and were they a suitable population?*	
*•* Participant demographic characteristics	Employee questionnaires
*•* Participant health information: perceived impact of work on health, previous experience of workplace health checks	Employee questionnaires
*•* Information on those who did not participate	Data collected at event
Effectiveness	
*Was the intervention successful, what was the impact of testing?*	
*•* Uptake of HIV tests, characteristics of those who had tests	Data collected at event Employee questionnaires
*•* Details of other health checks delivered	Data collected at event
*•* Reported employee learning from event	Employee questionnaires Manager questionnaires
*•* Information on acceptability and usefulness of HIV testing	Employee questionnaires Manager questionnaires
*•* Information on acceptability and usefulness of health checks	Employee questionnaires Manager questionnaires
Adoption	
*How successful was the intervention in recruiting the right companies/locations for the intervention?*	
*•* Summary data and representativeness of participating companies	Manager questionnaires
*•* Information about companies which were approached but did not participate	Interview with lead event organiser
*•* Recruitment processes and challenges	Interview with lead event organiser
Implementation	
*How, and how well, was the intervention delivered?*	
*•* Delivery of the intervention as intended	Interview with lead event organiser
*•* Experiences of attending the event	Employee questionnaires
*•* Views on the organisation of the event	Manager questionnaires
*•* Views on the scope of the event	Employee questionnaires Manager questionnaires
*•* Event costs	Interview with lead event organiser
*•* Online toolkit	Manager questionnaires
Maintenance	
*Has employer behaviour changed? How likely is that that they would participate again?*	
*•* Intention to include sexual health in future activities	Manager questionnaires
*•* Willingness to participate in future events	Manager questionnaires

## Results

**Fig. 1:**
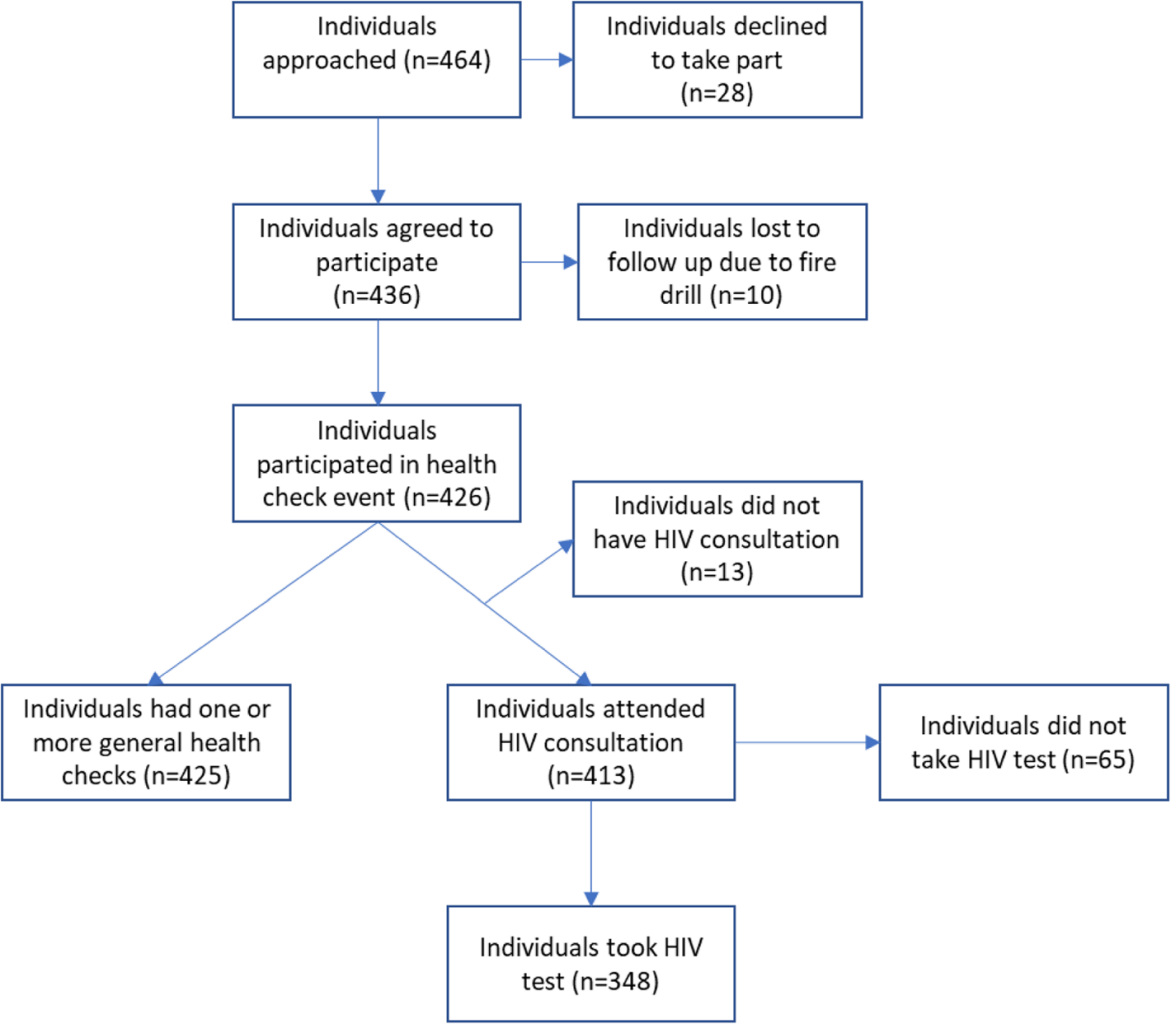
Summary of participant numbers through the study

A total of 426 individuals participated in health checks at 21 events, the number of participants per event ranged from 8 to 34. A further 10 individuals agreed to participate but left the event prematurely as the result of a fire drill and were therefore lost to follow up. Twenty-eight individuals declined to participate, 6% of those approached by the research team. Figure 1 summarises the numbers participating at different stages of the health check events.

Questionnaires were completed pre-and post-event by all participants (100% completion rate). Fifteen managers completed questionnaires across the 21 events (one of them had responsibility for multiple sites and gave a single response in relation to these; two managers did not complete questionnaires).

### Reach

Overall, health check participants were broadly representative of the wider construction workforce, with the main differences being a higher proportion of women in this sample, and a higher proportion of permanent workers. Workers largely considered themselves to be in good health. Many had never had a health check at work, and most considered that their work had an adverse impact on their health.

#### Participant demographic characteristics

Table 2 shows the details of those who participated in health checks. It also examines whether the participant population was representative of the wider construction workforce.

**Table 2:** Demographic characteristics of health check participants

	Male n = 348 (81.7%)	Female *n* = 78 (18.3%)	Total n = 426 (100%)	UK construction workforce, for comparison	*p*
	n (%)	n (%)	n (%)		
**Gender**				[75]	***p < 0.001***
	81.7%	18.3%		Male: 87.6%; female: 12.4%	
**Age (years)**				[76]^a^	*p = 0.233*
17–30	90 (25.9)	16 (20.5)	106 (24.9)	Age 16–24: 10%	
31–40	99 (28.4)	23 (29.5)	122 (28.6)	Age 25–49: 61%	
41–50	85 (24.4)	18 (23.1)	103 (24.2)	Age 50–64: 26%	
51–70	74 (21.3)	21 (26.9)	95 (22.3)	Age 65+: 2%	
**Ethnicity**				[77]	*p = 0.220*
White	313 (89.9)	62 (79.5)	375 (88)	White 87.7%	
Mixed	4 (1.1)	3 (3.8)	7 (1.6)	Mixed 1.2%	
Asian or Asian British	22 (6.3)	5 (6.4)	27 (6.3)	Asian/Asian British 6.6%	
Black or Black British	7 (2)	6 (7.7)	13 (3.1)	Black/Black British 3.0%	
Other ethnicity	0 (0)	1 (1.3)	1 (0.2)	Other 1.6%	
Not stated	2 (0.6)	1 (1.3)	3 (0.7)		
**Country of birth**				[27]	***p < 0.001***
UK	309 (88.8)	63 (80.8)	372 (87.3)	UK 90%	
Europe	14 (4.1)	7 (9.2)	21 (5)	Europe 7%	
Rest of world	20 (5.8)	6 (7.9)	26 (6.2)	Rest of world 3%	
Not stated	5 (1.4)	2 (2.6)	7 (1.6)		
**English as first language**				^b^ [78]	*p = 0.231*
Yes	326 (93.7)	71 (91)	397 (93.2)	92.3%	
No	19 (5.5)	7 (9)	26 (6.1)		
Not stated	3 (0.9)	0 (0)	3 (0.7)		
**Nature of employment**				[79]	***p < 0.001***^***c***^
Permanent	230 (66.1)	64 (82.1)	294 (73.7)	Men 56.2%; women 86.8% Total 59.9%	
Non-permanent (agency, contact, self-employed, other)	95 (27.3)	10 (12.8)	105 (26.3%)	Men 43.8%; women 13.1% Total 40.1%	
Not stated	23 (6.6)	4 (5.1)	27 (6.3)		
**Sexual Orientation**				^b^ [80]	***p = 0.052***
Heterosexual	337 (96.8)	78 (100)	415 (97.4)	Heterosexual 94.6%	
Homosexual	2 (0.6)	0 (0)	2 (0.5)	Homosexual 1.4%	
Other	2 (0.6)	0 (0)	2 (0.5)	Other 1.5%	
Not stated	7 (2)	0 (0)	7 (1.6)		

^a^ statistical comparison is against published data from the UK Annual Population Survey, this uses different age boundaries to this study

^b^ comparison is with total UK population, not construction workforce

^c^ the difference is significant for total population, and also for men only

Health check participants were representative of the wider construction workforce in terms of their ethnicity, age and whether English was their first language. There were more non-Europeans in the study sample than in the UK construction workforce overall (*p < 0.001*), but the proportions of UK versus non-UK workers did not differ significantly.

The 47 individuals who were born outside of the UK were from a total of 18 different countries, most commonly India (*n* = 11) and Jamaica (*n* = 4). Eleven individuals (6 men, 5 women) were born in a country where the prevalence of HIV is 0.5% or higher [81].

Participants were more likely than the wider construction workforce to be employed in permanent roles. There was weak evidence that participants were more likely to be heterosexual than the UK population in general - this may reflect reticence on this matter given the hegemonic masculinity evident in the construction sector [82]. The participant population had a higher proportion of women than the UK construction workforce, this is likely to reflect the fact that several study locations had a large administrative workforce as they were offices which were a work base for peripatetic tradespeople rather than being active construction sites.

Participants were not asked whether they lived away from home whilst they were working on this project, but they were asked to provide their home postcode. Six participants (out of 392 who answered the question) gave home postcodes which were over three hours’ drive from the site and were therefore assumed to have been living away from home. A further four participants were living two hours’ drive from the site. It is therefore estimated that up to 2.5% of participants could be classified as living away from home.

#### Prior experience of health checks

Participants were asked whether they had ever had a workplace health check before. Of 424 respondents, 181 reported that they had (42.7%) and 57.3% had not. Likelihood of having had a health check at work increased with older age (*p < 0.001);* only 25.5% of those aged 30 years or below had previously had a health check at work. The likelihood of having had a health check at work also varied by nationality, with migrant workers from Europe being less likely (19% reported a health check) and those from the rest of the world more likely (61.5%) to report having had a workplace health check previously compared to non-migrant workers *(p = 0.014).*

#### Participant health

The majority of participants reported themselves as being in good health – when asked ‘how healthy do you feel now?’, on a scale of 1–10, the median score was 8 (interquartile range 7–8), with 78.6% percent of responders scoring themselves at 7 or above, and 21.4% (91 individuals) scoring 6 or lower. Women reported worse health (median = 7) than men (median = 8), (*p = 0.07)*. Permanent employees reported worse health (median = 7) than those that were non-permanent (median = 8) (*p = 0.002)*, this difference persisted when the comparison was drawn for men only.

Participants were asked if work affected their health. Of 418 responses (8 missing), 22.5% (*n* = 94 participants) reported that their work affected their health a lot and 59.3% (*n* = 248) reported that it affected their health a little. The likelihood of reporting work-related health effects was higher for those who worked in permanent roles (252 workers, 87% of permanent workers who answered the question) compared to those who were non-permanent (70 individuals, 68.6% of those answering) (*p* < 0.001). There were no other significant interactions.

Table 3 shows the main work-related health issues reported by participants.

**Table 3:** Work related health issues reported by participants

Health effect reported	Number of participants (n = 426) (% of health check population)		
	Work affects my health a little	Work affects my health a lot	Total
Stress, pressure and mental health	80 (18.8%)	38 (8.9%)	120 (28.2%)
Musculoskeletal disorders, aches and pains	44 (10%)	21 (4.9%)	65 (15.2%)
Lifestyle impact e.g. overweight, sitting down too much, not being able to eat the right food or exercise because of work	28 (6.6%)	10 (2.3%)	38 (8.9%)
High workload	16 (3.7%)	9 (2.1%)	25 (5.9%)
Tiredness or fatigue	14 (3.3%)	7 (1.6%)	21 (4.9%)
Dust, breathing	9 (2.1%)	1 (0.2%)	10 (2.3%)
Other work hazards e.g. noise, vibration	3 (0.7%)	2 (0.5%)	5 (1.2%)
Positive impact of work	2 (0.5%)	2 (0.5%)	4 (0.9%)

Although *Reach* was not specifically addressed in the open questions, a small number of respondents commented on the importance of health checks for the construction population, either because of the nature of their health, or because they would not seek health care through other routes such as GPs, comments included: “*This is a fantastic chance to have on-site as most men don’t even bother with the doctors”* (Male worker); and “*Some people probably find it difficult to have time to get to the doctors”* (H&S manager).

#### Who didn’t participate?

The total number of workers who were on site on each day and therefore entitled to participate could not be ascertained. Although managers were asked to confirm the size of the site workforce (this information is shown with company details in Additional file 4), most only gave figures for those who were directly employed and did not include workers who were subcontracted or self-employed. Other sites were a base for peripatetic workers who attended briefly only at the beginning and end of the working day.

Most available appointments were filled at the events, although there were sometimes slow periods initially, particularly where advance promotion had been poor, followed by busier times as word spread. At some events, demand exceeded capacity, and additional events were arranged.

Twenty-eight individuals, at seven events, were approached by the research team but declined to participate in health checks. The most common reason given was being too busy (*n* = 14), this included three who were paid by work output (hence attendance would have had a financial impact). Other reasons given were having health checks elsewhere (*n* = 5), having unrelated health issues (*n* = 3) and perceiving they were too young to need a check (*n* = 2). At fourteen events, all appointments were allocated in advance of the event, so reasons for non-participation were not available.

### Effectiveness

Overall, participation in HIV testing amongst those attending for health checks was very high. Acceptability of HIV testing in the workplace environment was very high, with benefits for both workers and employers reported. There was good agreement that guidance given was understandable and high confidence of making lifestyle changes following the event.

#### Participation in HIV testing

Out of the 426 participants who attended for a health check, 413 (96.9%) opted to have a consultation about sexual health and 348 (81.7%) took an HIV test. Of those who took an HIV test, only 78 (22.4% of those tested) had previously been tested, and only 22 (6.3% of those tested) of these had been tested in the last 12 months. All HIV tests were non-reactive (negative). Eleven individuals were referred to a local sexual health clinic for further assessment or treatment.

No information was gathered on why participants declined a consultation about sexual health or an HIV test, as this information was available only to the sexual health teams to preserve participant confidentiality.

There were no differences in characteristics between participants who had an HIV consultation and those did not. Those who took an HIV test differed from those who did not in terms of age (*p = 0.043)*. Participants aged 31–40 were more likely than other age groups to take an HIV test, this is shown in Table 4. There were no other significant interactions.

**Table 4:** Participants who opted for an HIV consultation and/or HIV test, by age

		Had HIV consultation				Took HIV test			
		Yes		No		Yes		No	
Age group	n	Number	%	Number	%	Number	%	Number	%
17–30	106	103	97.2	3	2.8	86	81.1	20	18.9
31–40	122	117	95.9	5	4.1	109	89.3	13	10.7
41–50	103	101	98.1	2	1.9	82	79.6	21	20.4
51–70	95	92	96.8	3	3.2	71	74.7	24	25.3
Total	426	413	96.9	13	3.1	348	81.7	78	18.3

#### Acceptability and impact of HIV testing

Participants were asked whether they considered it acceptable to include HIV testing as part of a workplace event. Of the 421 participants who responded (5 missing), 408 (96.9%) said it was acceptable, while 13 individuals (3.1%) considered that it was not acceptable. Nevertheless, all 13 had chosen to attend the health check event, 11 of these thirteen individuals attended an optional HIV consultation at the event, and six then opted to take the HIV test.

The managers who had organised or overseen events were also asked about the acceptability of HIV testing in this context. All except one considered that it was acceptable. Reasons given included the potential to increase workers’ knowledge in this area, and the fact that it was “*Good to try to get rid of the stigma” (*H&S manager*).* The fact that uptake of HIV testing had been good was seen by several as confirming its value and acceptability, “*as take-up was high, colleagues obviously felt it was needed*” (Wellbeing manager).

One manager and two employees made negative comments about opt-in HIV testing at work, such as*, “I don’t feel this added value to the workers, the feedback was it felt awkward”* (H&S manager*)*, and also raised concerns about privacy when being given results in the workplace.

#### Acceptability, and impact of overall health check event

Participants found the event to be a positive experience. In response to the question ‘Did you feel that health information was given to you in a language and format that was easy to understand?’ of the 424 who answered the question (2 missing responses), 421 (99.3%) answered ‘yes’. Also, out of 422 who answered the question ‘Would you attend this kind of workplace health event again?” (4 missing), 419 (99.3%) said they would, and only three (0.7%) said they would not.

Further evidence of impact comes from responses to the question, ‘Did you learn anything new about your health in this event?”, 311 participants (74% of those responding, 6 missing responses) confirmed that they had. The likelihood of this did not vary significantly by age, ethnicity, gender, being UK born or speaking English as a foreign language. When asked ‘Do you intend to make any changes to the way you manage your health following this event?’ 296 (70.1%) participants confirmed that they did. Confidence that these changes were achievable was high, with a median score of 8 (IR 7–9) in response to the question, ‘on a scale of 1–10 how confident are you that you can make these changes?’ There were no significant differences between participant sub-groups in terms of their confidence in making changes.

Responses in the manager questionnaires supported these findings; 100% (*n* = 15) agreed that the health check events had been useful and that the events had been appropriate in terms of content, activities and focus. Perceived benefits included the positive impact on employee knowledge, the potential for improved health or to have provided reassurance. Comments included, “*it has provided them with useful health information that should help them keep their health under control*” (Site manager); “*It got all of the participants talking about health issues for the day*” (H&S manager); and, “*it helps to raise the awareness of employee health and wellbeing for both our employees and subcontractors*” (H&S manager).

Several managers reflected on the willingness of employees to attend and how well received the event had been, for example that it had been “*received very well from all operatives and staff that attended”* (H&S manager). Some also made comments about the positive benefits of the events for the employer. This was partly about being able to share information with other parts of the business, but there were also reputational benefits, and potential recognition through formal schemes for participating in the events, “*From a site perspective it boosted site morale showing that we were supplying on site health checks, the checks also featured in our Considerate Constructors Audit and was considered an innovative activity due to the research element of the testing*” (Site manager); and *“(it) helped show we are supporting health awareness with the workforce*” (H&S manager).

### Adoption

Overall, participating companies were mostly large or very large, and therefore are not representative of the construction landscape; although participants were also drawn from their sub-contractors who are likely to be smaller companies. Recruitment of companies was challenging and time consuming.

#### Participating companies

A total of 21 sites took part in the research, from ten companies. Two further companies had been recruited and had scheduled events, but these were cancelled due to the COVID-19 pandemic. Details of the companies/sites which participated are presented in Additional file 4.

For all but one company, there were reports of existing health or wellbeing activities, including a combination of individual interventions, (health checks, health kiosks), team events (‘tough mudder’, charity events) and organisational programmes (mental health first aid, employee assistance programmes, private health care, health promotion such as smoking cessation, healthy eating). For two companies, occupational health resources were mentioned (but did not specify health surveillance). No companies included sexual health awareness or HIV testing in their existing wellbeing activities.

#### Representativeness of companies

Seven out of the ten companies participating in the research had over 1000 employees, fewer than 0.03% of UK construction companies are this size [83]. They are therefore not themselves typical of UK construction companies: 96% of UK construction companies employ fewer than fourteen workers. However, all workers on the study sites were eligible to participate in the research, including those employed by the lead company which was responsible for the site, and also those working on behalf of sub-contractors, which might be small to medium enterprises (SMEs), or micro-sized companies. Therefore the companies and their supply chain taken as a whole are likely to be more representative of the construction landscape than the named companies participating, although the data available do not allow this to be examined in detail.

The participant companies varied in terms of the proportion of their workforce which was female. Some employed fewer than 1% women on site, others were office-based locations and had a much higher proportion of female employees. Again, this is not representative of the wider construction sector, and is likely to account for the high proportion of female participants taking part in the research.

#### Experiences of recruitment

Initial recruitment was through email contact with companies which were identified through internet searches or from the UK Construction Index. Initially, the focus was specifically on construction companies, this was expanded to included construction-related companies to increase the scope, as the response rate was initially low. A total of ninety-six companies were approached to gain access to the twelve who agreed to participate (12.5%) (events with two of these companies were cancelled due to the COVID-19 pandemic). The main challenge was gaining access to a named individual who had the responsibility and authority to discuss the research and most contacts stalled at this early stage. Commonly, initial contact points (such as an *info@* email address) failed to pass a message on, or initial contacts were made and then a company representative declined to participate in further discussion.

In 21 companies, the lead researcher made contact successfully and explained the research, but the company decided not to proceed. The most common reasons given included perceived overlap between the research and general health checks already provided (six companies) - albeit none providing HIV testing; the company perceiving themselves to be too small (three companies); and the company not having active sites within the geographical area of the study (four companies). Four companies simply withdrew from discussions or stopped answering calls. The inclusion of HIV testing in the research was not discussed until companies had expressed a desire to participate. One raised some initial concerns about it, and required further supporting information, but was then happy to proceed. No other companies raised any issues or concerns about the inclusion of HIV testing.

Key learning during the recruitment phase included the value of persistence, and the importance of getting to the right person to secure the sites and events.

### Implementation

Overall, implementation was successful, with all events being conducted as planned. The success of events was enabled by a high degree of flexibility at the planning stage and the skills of the health care staff involved.

All 21  events were delivered successfully, with general health checks and HIV tests provided at all. Three themes related to implementation were identified from the responses to open questions in the managers’ and employees’ post-event questionnaires.

#### Event organisation

Events were seen as being successful because they had been well planned in advance, with negotiation and flexibility between the researchers and the host site. It was identified as important that events were scheduled for times when the workforce were most available.For example, at those stages of the project when there were high numbers on site, allowing good participation. Arranging multiple visits to a site was another way of achieving this. “*Our two visits were held at the perfect times, as the first caught our demolition operatives just before they were leaving site at the end of December, and the second visit then caught the new starting operatives in January giving a good range of responses from operatives in very different areas of the industry*” (Site manager). One manager also commented on the benefit of scheduling to tie in with other motivators such as “*post-Christmas, back to work since the new year / new me*” (H&S manager). Ensuring capacity throughout the working day was also important to ensure that workers could attend without the event interfering too much with their usual duties.

Challenges encountered by the research team in running events were mostly linked to poor organisation on the part of the host company such as failing to book suitable rooms for testing or failing to promote the event to workers well in advance. Whilst inconvenient, these issues were always successfully resolved on the day.

#### Positive experiences

Workers who responded to an open question about whether there was anything else they would like to add commented mostly about their positive experiences of the event. In particular, they noted the efficiency and professionalism of the health check teams, their friendliness, and their knowledge and ability to make the topics understandable. Comments included that the event had been, “*well delivered, friendly, knowledgeable”* (Male worker); that *staff "were friendly and made it all easy to understand”* (Female worker); and even, *“excellent staff, worth making the trip on my day off”* (Male worker). Similar comments were made by managers.

#### Testing scope

The third theme identified was the scope of testing. Several managers commented that it would be useful to include additional tests, such as those for diabetes, cholesterol, body fat and lung capacity. This view was supported by participants, who were asked specifically if there were other health assessments they would be interested in for future events. Out of 415 who answered the question, 167 (40.2%) answered ‘yes’. The most popular tests to include in future events related to cholesterol (34 participants), diabetes (28 participants), and mental health issues (15 participants). There were also 15 requests for assessment related to specific work-related hazards such as hand arm vibration, noise and dust, and six related to musculoskeletal disorders.

#### Online toolkit

The managers were asked whether the Test@Work toolkit [66] had been a helpful resource. Those that answered this question (14/15) felt it had been useful. Some commented on how user-friendly it was, others on how it had enhanced their knowledge and awareness of workplace health promotion, health checks and HIV testing. Although the toolkit was designed to increase the managers’ knowledge so that they could promote the health check event effectively with the workforce, some shared it directly with workers. Others shared it with other colleagues in the company to increase awareness more widely.

#### Costs

The direct costs of the overall health check were low, as volunteers gave their time free of charge. This involved a major effort to recruit, train and manage teams of volunteers attending each event, but also provided significant benefits for these healthcare trainee volunteers in terms of professional development [69]. The health check team spent a total of 107 person-days on work sites to deliver the 21 events (37 for HIV professionals, 70 for health check volunteers), with 3–7 people at each event. Equipment for health checks (e.g., sphygmomanometer, scales, height and waist measures) cost approximately £300. For HIV testing, the cost was nominally £50 per person tested (for test kit and staffing). On some sites, testing was funded by healthcare outreach services.

There were no direct charges to the construction sites for participating, but they had to absorb the opportunity costs of time when workers were attending the event rather than doing their usual jobs. Each individual attended the event for around 30–45 min, although the time could increase if there was extra waiting time. No participating companies objected to this, although it was not clear whether the lost time was at the expense of the lead company or the sub-contractor. Three workers declined to attend because they were concerned about losing earnings as a result of reduced output for the day, and others might also have been discouraged from signing up to the programme for this reason, particularly those who were sub-contracting or self-employed. A small number of workers were initially reluctant to attend but did so once given reassurance that they would not be penalised.

### Maintenance

Overall, there was a high willingness from companies to participate in future events which were arranged by an external partner. The likelihood of companies incorporating HIV awareness in their own events was limited by an organisational focus on other topics.

Managers who hosted events were asked whether they were planning to incorporate sexual health and HIV awareness in their own workplace health programmes in future as a result of their involvement in Test@Work. One organisation confirmed they had already taken action to implement change and five further organisations confirmed future implementation intentions -“*Sexual health has now been added into our induction presentation in order to highlight its importance, we have also issued a toolbox talk on HIV awareness which was issued last month”* (Site manager). Nine respondents were not planning to take the topic forward (two did not answer), this was generally because it did not fit with their company’s pre-existing campaigns which were focused mostly on mental health. Some managers shared that they did not have the authority to make the decision as health programmes were planned centrally (e.g. organisational-level) rather than locally (site-level).

In terms of participation in future events, all managers who completed a post-event questionnaire confirmed that they would host such events again if there was an opportunity. Eight of the 10 companies involved in the research had already undertaken or committed to additional events before the project ended.

## Discussion

### HIV testing in construction

This study found opt-in HIV testing in UK construction workplaces to be very successful. HIV testing was acceptable to workers and uptake was high, with almost all workers at our health check events choosing to attend a sexual health consultation, and 8 in 10 opting in for an HIV test. This compares favourably with other HIV testing rates outside of traditional settings, such as workplaces in Africa and Afghanistan (67%) [11], community settings in Europe (9–95% testing uptake is reported, but most studies evaluated have uptake below 80% and provide incomplete data on numbers invited to test) [12], and new registrants at UK General Practices (45% uptake of testing offer) [84]. The proportion who were being tested for the first time in our study (78%), was far higher than reported in other community settings (e.g. 14% first time tests in a systematic review of testing in resource-rich countries) [12]. This is important, given the association between first time testing and higher rates of seropositivity [11].

No cases of HIV were identified by this study, but this does not negate the value of testing, particularly given the relatively small size of the study population. Seropositivity for HIV testing is 0.4% in UK community settings overall (with most testing targeting high risk groups such as MSM) [1], and has been reported at 0.2% in a cohort of new GP registrants [84]. The likelihood of case finding could be increased by targeting potentially higher risk construction populations, such as workers living in London or working on major projects. London construction projects employ four times as many European migrant workers and more than twice as many non-EU migrant workers compared to UK construction overall [27], with similar variations in ethnicity. During construction of the Olympic park for London 2012 for example, 24% of the workforce were of BAME ethnicity, compared to 7% of the UK construction workforce at that time [85]. Additionally, London based projects and mega projects are more likely to employ workers who are living away from their families [29, 86], with the associated risk behaviours reported in these populations [30, 87]. The combination of high uptake of testing (as achieved in this study) with likelihood of higher prevalence of HIV would make this a valuable approach to identifying undiagnosed cases of HIV. Regular testing (e.g. every three years) has been identified as both desirable and cost effective even where incidence is as low as 0.01% pa [88], and annual testing is likely to be cost effective once prevalence reaches 0.8% [89].

It is worth noting that cases could have been identified in our sample after the health check events since workers who did not have an HIV test on the day were given information at the time about how to access free HIV testing at their convenience. Also, 291 construction workers from the Test@Work study (68.3% of health check participants) signed up for an additional text messaging intervention called Test@Work Texts [90] that was delivered after their health check event, between March – June 2020 and during the COVID-19 pandemic when access to sexual health clinics was limited. Workers received a series of text messages promoting sexual health and HIV testing (as well as other areas of health) over a 10-week period, with subsequent text-based evaluation of the intervention. A small number of respondents reported having taken a further HIV test after the event. It is therefore possible that positive cases may have been identified in our sample outside of the health check event days.

Educating the workforce and raising awareness of the importance of HIV prevention and testing are important, as this could influence a far higher proportion of workers than can be reached through on-site testing. This could be addressed through further implementation of the Test@Work online toolkit [66] particularly if it can be introduced through major construction projects, companies or industry bodies. Individual sites can then follow this up by liaising with local testing providers through the healthcare services. Signposting to self-testing services might also be helpful in some circumstances [91]. As with onsite testing initiatives, educational interventions would be particularly valuable if they targeted those workplaces with a higher potential HIV prevalence. However, those who are most at risk of HIV might be the least likely to seek testing due to fear of negative consequences or stigma, particularly if there are concerns about being able to keep the diagnosis confidential at work. Design of interventions needs to take this into account.

### General health checks in construction

HIV tests in this study were provided as part of a wider health check. This is recommended in the literature as a way of normalising HIV testing and reducing stigma [15, 60, 92, 93] - the high uptake of testing in this study supports this approach. The study also illustrated the direct benefits of offering these wider health checks. They were highly valued by workers who participated and managers who hosted them, with high levels of interest expressed towards arranging further events. Encouragingly, our data show that many workers planned to make lifestyle changes to improve their health and felt confident that they would be able to do so. This is important given the recognised poor health of the construction workforce [40, 46, 49], and the ‘*anti-health promoting’* nature of the construction industry [94]. Although there are national health programmes which have a similar scope to the general health checks offered in our study, their uptake is often limited. For example, the UK National Health Service (NHS) health check is offered to those aged between 40 and 75 in the UK [95] but uptake is only just over 50% [96]; and uptake is lower amongst men and those at the younger end of the target range [97, 98]. A particular barrier to men participating in such programmes is lack of time to do so [99], making workplace events such as this especially helpful for this population.

However, there are also challenges when conducting health interventions in the construction sector. It is an industry which is highly fragmented, with extensive sub-contracting, high levels of self-employment, and an excessive focus on cost [100]. In the current study, the majority of participating companies were extremely large and self-employed workers were underrepresented, only 26% of those seen were self-employed compared to 40% in the wider construction population. This mirrors the findings of Hanna et al. [94], that health promotion in construction is more likely to occur with large employers and those with greater numbers of directly employed workers, as others are unlikely to be able to see sufficient benefit to offset the costs.

Interventions like the one described here, which are open to all workers on site regardless of whether they work for the main contractor or its subcontractors, are one way of redressing this imbalance. This approach has been taken by UK mega projects such as London 2012 [101] and Heathrow Terminal 5 [102] to improve the health of workers through the supply chain. It can also help knowledge and good practices to ‘trickle down’ through the industry [103]. However, further efforts are needed to access those SMEs and micro-organisations that are not contracted on larger projects; and to improve access to health promotion for self-employed and agency workers who are paid on the basis of the measured work that they complete [104] and do not wish to lose working time.

Regardless of how successful health check events are, the likelihood of workers making long term changes to their lifestyle is influenced by the wider context including family, workplace and industry factors [105–107]. There was evidence of this in the current research, with participants making comments about the difficulties of healthy eating and exercise due to the constraints of work demands. Additionally, over 80% of participants considered that work had a direct adverse impact on their health. The most frequent conditions mentioned were musculoskeletal disorders and stress/mental health issues, which are the most prevalent work-related health problems reported in UK construction [108]. Any approach to improve worker health needs to take this context into account. For example, the Test@Work online toolkit [66] used successfully in this research to inform managers about the value of HIV testing could be developed further to highlight to employers the importance of structural measures to improve the health of their workforce, alongside measures that individuals might take themselves.

The non-HIV element of the Test@Work intervention focused specifically on general health, those factors which are typically influenced by lifestyle, such as stress, diet, exercise and smoking. However, some participants raised issues which were broader than this. For example, when asked what additional health checks would be valued, some mentioned tests of lung function, hearing or hand arm vibration syndrome. These are examples of health surveillance, which are a legal requirement for those who are exposed to particular work hazards. Although the number that specifically mentioned this was small, the fact that more than half of participants confirmed they had never had a workplace health check confirms the limited access to Occupational Health (OH) services in this population. This is a known challenge. Across the UK, only 14–18% of the workforce have access to OH services [109, 110]. The proportion is typically even lower in construction, despite the higher health risks, due to the way the sector is configured and costed and the high proportion of SMEs [111, 112] . General health checks to support lifestyle changes, and occupational health provision to address work related conditions are both important. It is important that employers and workers understand the difference, and recognise that one is not a substitute for the other. Again, this could be highlighted in revised versions of the online toolkit.

### Recommendations for future testing and additional interventions

Recommendations for future events of this nature are summarised in Table 5.

**Table 5:** Recommendations for future interventions

Recruiting companies for workplace health interventions	
• It is important to identify named contacts when targeting companies. Local groups of H&S professionals (such as IOSH in the UK) may offer a route for this, snowball recommendations between companies are also useful	
• A greater focus on SMEs and micro-organisations is important, particularly those that do not work on major projects. They may be accessed through industry forums such as Working Well Together and Build UK	
• It is important to improve access to workplace health promotion for self-employed workers, this might be achieved by offering appointments at the beginning and end of working days. Providing links to resources that can be accessed outside of work hours might also be important for this group e.g. online resources, self-testing opportunities or weekend/evening clinics	
HIV testing in construction	
• There is an expected benefit of targeting higher risk populations e.g. in major cities and on mega projects, where a greater proportion of workers may be from higher risk countries and/or living away from home and more likely to demonstrate high risk behaviours	
• Events must be planned to ensure reactive results can be sensitively and discreetly managed, and to demonstrate to participants that this can happen	
• Options should be available for individuals to have testing independently after the event if they prefer	
• Wider promotion through the sector is needed to get sexual health and HIV on the agenda of major organisations and projects and the industry more widely	
• HIV testing could be driven by outreach from public health services, to enable central funding of testing	
Providing general health checks	
• Events should be planned to coordinate with and promote existing OH arrangements	
• The ‘volunteer’ model for health check delivery proved very successful, and reduces costs of health checks, but carries high administrative, supervisory and support requirements	
• Health checks could be expanded to include tests such as cholesterol and diabetes which are part of the NHS health check in the UK	
Test@Work online toolkit	
• Education of managers is useful to raise awareness of HIV, so that they can inform and support their workforce and promote testing	
• A separate resource for workers provided in advance of health checks would be useful e.g. a one-page document or online resources	
• There is an opportunity to remind employers of their legal obligations in respect of occupational health checks in addition to general health checks which focus on lifestyle factors	
• There is an opportunity to educate employers on the importance of organisational changes to improve worker health and support lifestyle changes	

### RE-AIM – value of structured evaluation

Using the RE-AIM tool was an effective way of evaluating the intervention, particularly in considering the representativeness of participant individuals and companies. This helped to highlight the particular challenges of working in the construction sector, and how these might be addressed.

### Limitations and further research

This study achieved its aim of delivering and evaluating opt-in HIV testing in construction workplaces; the similarities between participants and the wider UK construction workforce give confidence that the study sample was broadly representative of this population. However, because workers on a construction site typically come from a wide variety of companies and agencies, it was not possible to directly compare health check participants to others on site who chose not to attend. This makes it more difficult to understand the barriers to attending for HIV testing (and general health checks) in construction workplaces and how these might be overcome. Further work is in progress to explore this.

## Conclusions

Test@Work is the first study to deliver and evaluate opt-in HIV testing in the UK construction sector. We found strong evidence that HIV testing can be implemented successfully through construction workplaces, with high uptake and high numbers of first-time tests compared to other community settings. Further studies are required to confirm whether the prevalence of HIV in this population make testing of this nature an effective and cost-effective approach. Testing would have the most impact if it targeted companies with more high-risk workers e.g. those in major cities or on mega- projects.

There was strong evidence regarding the desire for and acceptability of general health checks for this population, despite initial challenges in negotiating access within organisations. Testing was largely conducted on sites run by very large companies. This illustrates the importance of this route to access the wider construction workforce given the fragmented nature of the sector, but other approaches are also needed to reach workers in small and micro companies, and those which do not work on major projects.

Education regarding HIV testing and general health risks are important to support workplace testing initiatives. The Test@Work online toolkit was a useful approach and could be further developed to ensure employers are aware of the wider obligations and opportunities in respect of worker health.

Finally, given the high levels of acceptability of HIV testing in the workplace, our study suggests that this is an approach that may also be particularly valuable in countries with higher HIV prevalence rates in the general population.

## Supplementary Information

**Additional file 1.** Participant pre-and post-event questionnaires.

**Additional file 2.** Manager pre-and post-event questionnaires.

**Additional file 3.** Sexual Health_HIV Consultation Recording Sheet.

**Additional file 4.** Details of companies and sites visited.

## Data Availability

The Test@Work study does not have ethical approval to share the study dataset for reasons of participant confidentiality. Some of the study data may be available from the corresponding author on reasonable request.

## Abbreviations

ART: Antiretroviral therapy
BAME: Black, Asian and minority ethnic
BMI: Body Mass Index
CCGs: Clinical Commissioning Groups
EEA: European Economic Area
EU: European Union
GP: General Practitioner (family doctor)
MSM: Men who have sex with men
H&S: Health and Safety
HIV: Human Immunodeficiency Virus
NHS: National Health Service
PHQ-2: Patient Health Questionnaire-2 item
SME: Small and Medium sized Enterprises (typically fewer than 250 employees)
UK: United Kingdom
WHO: World Health Organization
