# Supplementary material for: Test@work: evaluation of workplace HIV testing for construction workers using the RE-AIM framework
Source: BMC Public Health. 2021 Sep 24;21:1737. doi: 10.1186/s12889-021-11739-z (PMC8464147; doi:10.1186/s12889-021-11739-z)
Supplement: Supplementary file 1 — Additional file 1. Participant pre-and post-event questionnaires. [file 12889_2021_11739_MOESM1_ESM.docx]

**Attendee Registration and Feedback form**

**Please complete this page BEFORE your health check**

| Age |  |
| --- | --- |
| Gender |  |
| Country Of Birth |  |
| Ethnicity  White  A British  B Irish  C Any other white background  Mixed  D White and black Caribbean  E White and black African  F White and Asian  G Any other mixed background | Asian or Asian British  H Indian  J Pakistani  K Bangladeshi  L Any other Asian background  Black or Black British  M Caribbean  N African  P Any other Black background  Other Ethnic groups  R Chinese  S Any other ethnic group  Z Not stated |
| Current Home postcode |  |
| Sexual Orientation | Straight Gay Other |
| Are you a full or part time worker? |  |
| Are you a permanent worker or non-permanent (e.g. contract or agency worker?) |  |
| Is English your first language? |  |

**On a scale of 1-10, how healthy do you feel now?** (circle)

1 2 3 4 5 6 7 8 9 10

1=poor health 10=excellent health

**Have you ever had an HIV test before?** (circle) *Yes No*

**If Yes – how long ago was this?** (tick)

_____Less than 3 months ago

_____in last 3-12 months

_____1-2 years ago

_____over 2 years ago

This information is only for the purposes of generating statistics about the overall group of people attending our events. We do not store this information with your name, and by completing this you agree (in line with data protection laws) the information is to be stored on manual forms and computer files on the understanding that it will be held securely and accessed only by authorised personnel. If you provide your phone number, it will only be used for the purpose of sending you health promotion text messages over a single time period. After the project is completed all personal data is destroyed.

**Please complete this page AFTER your health check**

**Hand the form to any of our team.**

**Follow Up Text Messages**

All numbers given will be entered into a free prize draw.

We would like to offer you the opportunity to receive some text messages to follow up on some of the health/HIV information that has been provided in the event today. If you are happy to receive 15 text messages sent over a series of weeks in 2020, please provide us with your mobile number here.

[Mobile phone number………………………….........................].

You can unsubscribe from the messages or delete them at any time.

| Have you ever had a health check in your workplace before? | Yes / No |
| --- | --- |
| Did you learn anything new about your health in this event? | Yes / No |
| Did you feel that health information was given to you in a language and format that was easy to understand? | Yes / No |
| One part of the event today was an HIV testing service. Do you think it is acceptable to have this as part of a workplace health event? | Yes / No |
| Do you intend to make any changes to the way you manage your health following this event? | Yes / No |
| If yes, on a scale of 1-10, how confident are you that you can make these changes? 1=not at all confident, 10 = extremely confident. | Give a number 1-10 |
| Are there any other health assessments that you would be interested in? | Yes / No |
| *Which* other health assessments would you be interested in? | Name the health assessments |
| In what ways, if any, does your work affect your health? | Not at all/  A little/  A lot |
| What is affected? | Physical health/  Mental health/  Other (specify) |
| Tell us how? |  |
| Would you attend this kind of workplace health event again? | Yes / No |
| Is there anything else you would like to tell us or comment on? |  |

**Thank you very much for taking the time to complete this questionnaire**
