# Supplementary material for: Test@work: evaluation of workplace HIV testing for construction workers using the RE-AIM framework
Source: BMC Public Health. 2021 Sep 24;21:1737. doi: 10.1186/s12889-021-11739-z (PMC8464147; doi:10.1186/s12889-021-11739-z)
Supplement: Supplementary file 2 — Additional file 2. Manager pre-and post-event questionnaires. [file 12889_2021_11739_MOESM2_ESM.docx]

**Company: Pre-event Questionnaire**

**Tell us something about your workforce**

| **Question 1** | How many employees do you have? |  |
| --- | --- | --- |
| **Question 2** | How many are on agency contracts? |  |
| **Question 3** | How many are on permanent contracts? |  |
| **Question 4** | How many work full time? |  |
| **Question 5** | How many work part-time? |  |
| **Question 6** | How many men are in the workforce? |  |
| **Question 7** | How many women are in the workforce? |  |
| **Question 8** | What is the age range of your employees? | Lowest age:  Highest age:  Average age: |
| **Question 9** | What percentage of your employees are migrant workers? | % |
| **Question 10** | What is the ethnicity of your employees?  *White*  White British  White Irish  White other  *Mixed*  White and black Caribbean  White and black African  White and Asian  Any other Mixed  *Asian or Asian British*  Indian  Pakistani  Bangladeshi  Any other Asian background  *Black or Black British*  Caribbean  African  Any other black background  *Other ethnic groups*  Chinese  Any other ethnic group  Not stated | Number of employees |

**Workplace Health and Wellbeing**

| **Question 1** | Does the company currently provide workplace health and well being initiatives? | Yes No  Please tell us more…. |
| --- | --- | --- |
| **Question 2** | Do the company’s existing workplace health initiatives include HIV testing? | Yes No  Please tell us more…. |
| **Question 3** | Do the company’s existing workplace health initiatives include HIV awareness raising*?* | Yes No  Please tell us more…. |

**Thank you very much for taking the time to complete this questionnaire.**

**Company: Post-event Questionnaire**

Thank you very much for hosting the workplace health and well being events. We would like to ask a few questions to assess your views and experiences of the events.

| Were the health check events useful? | Yes No  Please tell us more…. |
| --- | --- |
| Were the events held at the right time? | Yes No  Please tell us more…. |
| Were the events appropriate in terms of content, activities and focus? | Yes No  Please tell us more…. |
| Did you find the online toolkit useful? | Yes No  Please tell us more…. |
| Was it acceptable to include HIV testing as part of the event? | Yes No  Please tell us more…. |
| Would you host such events again if provided by an external organisation? | Yes No  Please tell us more…. |
| Are you planning to incorporate sexual health and HIV awareness into your own workplace health promotion activity? | Yes No  Please tell us more…. |
| Will you use the online health screening toolkit in the future? | Yes No  Please tell us more…. |
| Please provide any further comments on the usefulness, acceptability and appropriateness of the health check events | Please tell us more…. |

**Thank you very much for taking the time to complete this.**
