# Supplementary material for: Test@work: evaluation of workplace HIV testing for construction workers using the RE-AIM framework
Source: BMC Public Health. 2021 Sep 24;21:1737. doi: 10.1186/s12889-021-11739-z (PMC8464147; doi:10.1186/s12889-021-11739-z)
Supplement: Supplementary file 3 — Additional file 3. Sexual Health_HIV Consultation Recording Sheet. [file 12889_2021_11739_MOESM3_ESM.docx]

|  | **Health Check Recording Sheet** |
| --- | --- |

**Date: Venue:**

| **Participant**  **ID** | **Gender**  **(M/F/Other)** | **Ethnicity/ Nationality  (please record the nationality of attendee from their reg sheet)** | **Date of Birth** | **Consultation Delivered**  **Yes/No** | **Test Delivered**  **Yes/No** | **Follow up/referral information** |
| --- | --- | --- | --- | --- | --- | --- |
|  |  |  |  |  |  |  |
|  |  |  |  |  |  |  |
|  |  |  |  |  |  |  |
|  |  |  |  |  |  |  |
|  |  |  |  |  |  |  |
|  |  |  |  |  |  |  |
|  |  |  |  |  |  |  |
|  |  |  |  |  |  |  |
|  |  |  |  |  |  |  |
|  |  |  |  |  |  |  |
|  |  |  |  |  |  |  |
|  |  |  |  |  |  |  |
|  |  |  |  |  |  |  |
|  |  |  |  |  |  |  |
